# Clinician Absences and Contributing Factors During a COVID-19 Surge: Potential Areas for Intervention and Planning

**DOI:** 10.5811/westjem.2021.11.52715

**Published:** 2022-02-14

**Authors:** Daniel Grahf, Jad Dandashi, John Deledda, Phyllis Vallee, Taher Vohra

**Affiliations:** Henry Ford Hospital, Department of Emergency Medicine, Detroit, Michigan

## Abstract

**Introduction:**

Our goal was to quantify healthcare clinician (HCC) absenteeism in the emergency department (ED) during the coronavirus disease 2019 (COVID-19) surge and to identify potential interventions that may mitigate the number of absences.

**Methods:**

This was a retrospective, descriptive record review that included 82 resident physicians, physician assistants, and staff physicians who were scheduled to work more than three clinical shifts during March 2020 in an urban, academic ED that received a high number of coronavirus disease 2019 (COVID-19) patients. Exposure was defined as a healthcare clinician who was not wearing appropriate personal protective equipment (PPE) having contact with a confirmed COVID-19 positive patient in the ED. The main outcome was the number of HCC absences secondary to exposure to or symptoms concerning for COVID-19.

**Results:**

During March 2020, of 82 ED HCCs, 28 (34%) required an absence from clinical duties, totaling 152 absentee calendar days (N = 13 women [46%]; N = 15 men [54%]). Median HCC age was 32 years (interquartile range 28–39), and median number of days absent was four (interquartile range 3–7). While 16 (57%) of the total absences were secondary to a known exposure, 12 (43%) were symptomatic without a known exposure. A total of 25 (89%) absent HCCs received COVID-19 testing (N = 5 positive [20%]; N = 20 negative [80%]) with test results returning in 1–10 days. Eleven (39%) symptomatic HCCs had traveled domestically or internationally in the prior 30 days.

**Conclusion:**

Emergency departments should anticipate substantial HCC absences during the initial surge of a pandemic. Possible interventions to mitigate absences include early and broad use of PPE, planning for many asymptomatic HCC absences secondary to exposures, prioritizing HCC virus testing, and mandating early travel restrictions.

## INTRODUCTION

Emergency medicine plays an essential role during pandemics and epidemics, especially during initial phases. Without enough healthcare clinicians (HCC) at this critical time, patient care may become compromised.^1^ This is also the time in which HCCs are most vulnerable to pathogen exposure.^2^ Predictive models have shown that during a pandemic, up to 25–50% of a physician workforce could be absent at any given time secondary to illness and exposure.^3^ One database showed that from March 1–May 31, 2020, HCCs accounted for approximately 6% of adults hospitalized with COVID-19.^4^

Other studies have shown an increase in HCC absenteeism correlating with the first weeks of the pandemic.^5^ In a study in England, physician absences more than doubled during a COVID-19 surge with greater than 50% of those absences related to COVID-19.^6^ There is a notable paucity of literature describing the effect that the initial phases of a pandemic have on emergency department (ED) HCC absences or the possible interventions that could curb the number of absences.^7^ This lack of data places frontline EDs at undue risk for inadequate HCC staffing at a time when patient care needs are greatest.^8^

The purpose of this study was to quantify HCC absenteeism in the ED during the outbreak of the novel severe acute respiratory syndrome coronavirus 2 (SARS-CoV-2) virus and to highlight the timing and factors associated with absenteeism. We conducted the study at an institution that experienced a substantial COVID-19 surge. Our goal was to provide insights for frontline departments where COVID-19 disease burden may yet occur and for potential future epidemics and pandemics.^9^–^11^

## METHODS

### Study Design and Setting

This was a retrospective, descriptive record review with a waiver of consent approved by the hospital institutional review board.

### Selection of Participants and Data

We conducted the study in an urban ED that receives over 100,000 annual patient visits. The ED is staffed by 127 HCCs including resident physicians, advanced practice providers, and attending physicians. Healthcare clinicians who worked more than three clinical shifts during March 2020 were included in this study. The department created an external database that tracked HCC absences secondary to COVID-19 exposures or symptoms during the COVID-19 pandemic to assist with ED staffing. The database was blinded and then analyzed by the research team.

### Definitions

Exposure was defined as a HCC without full personal protective equipment (PPE) having contact with a confirmed COVID-19 positive patient in the ED. Full PPE was defined as gloves, gown, eye protection, and mask. Surgical mask was acceptable except during any aerosolizing procedure, which then required a respirator mask. Symptomatic HCC screening varied over the study period, but primarily included fever, constitutional symptoms, respiratory symptoms, or gastrointestinal symptoms. Laboratory testing of nasopharyngeal swabs evaluated for the SARS-CoV-2 virus using polymerase chain reaction assay. Travel history was defined as any travel 30 days prior to exposure or symptom development.

### Timeline of Events

Our first case of COVID-19 was confirmed on March 10, 2020. All institutional international and non-essential domestic travel was cancelled on March 11. On March 12, testing criteria were changed from a more targeted approach (Figure S1) to a much broader approach (Figure S2). Triage changes were implemented on March 13 to begin screening patients for COVID-19 symptoms and risk factors prior to entering the ED. A dedicated testing area for stable patients was created on March 17. On March 18, full PPE became required for all patient encounters. Employee health allowed asymptomatic, exposed HCCs to return to work while wearing a mask on March 20, whereas previously HCCs had been placed on home quarantine for 14 days. All exposures were still required to be reported. Nasal swabbing by providers in full PPE in a dedicated testing area was implemented on March 21.

### Analysis

Descriptive statistics and graphical representations superimposed with explicit event dates were created using Microsoft Excel (Microsoft Corporation, Redmond, WA).

## RESULTS

### Study Subjects

During March 2020, there were 82 HCCs who worked more than three clinical shifts in the ED, and 28 (34%) who required an absence secondary to a COVID-19 exposure or symptoms for a total of 152 calendar absentee days. Of the 28 absences, 13 (46%) were women and 15 (54%) were men. The median age was 32 years (interquartile range [IQR] 28–39) and the median number of days absent was 4 (IQR 3–7).

### Main Results

**Figure 1:**
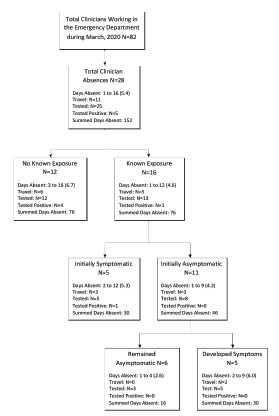
Breakdown of healthcare clinician absences* in the emergency department. *ED*, emergency department.

Of the total 28 HCC absences, 16 (57%) (N = 11 initially asymptomatic [69%], N = 5 initially symptomatic [31%]; N = 5 initially asymptomatic and went on to develop symptoms [31%]) were secondary to a known COVID-19 patient exposure and 12 (43%) were symptomatic without a known exposure (Figure 1). While 25 (89%) of those absent received COVID-19 testing, only five (20%) had positive results. Of those five who tested positive for COVID-19, four (80%) were symptomatic with no known exposure and one (20%) was symptomatic with a known exposure. A total of 11 (39%) HCCs with absences had traveled domestically or internationally, all were symptomatic, and four (36%) tested positive for COVID-19 (Figure 1).

There were 24 (86%) new HCC absences from March 10–21 compared to seven (16%) after March 21 until March 31. Of the HCCs with exposure-related absences, 80% occurred on March 13 and 14. Twelve HCCs who were initially symptomatic had a negative test result. The time their tests took to result ranged from 1–10 days. The eight HCCs with results in less than five days were absent for an average of 3.3 days. The four HCCs with results in five or more days were absent for an average of 6.5 days.

## DISCUSSION

**Figure 2:**
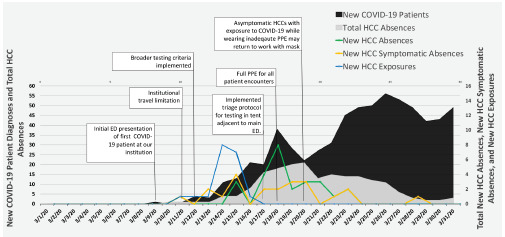
Healthcare clinician absenteeism and new patient diagnoses of COVID-19 in the emergency department. *HCC*, healthcare clinician; *COVID-19*, coronavirus disease 2019; *PPE*, personal protective equipment; *ED*, emergency department.

After the first institutional case of COVID-19 was diagnosed, there was a large burden of HCC absenteeism (Figure 2). Data has shown that this can be a burden for patient care, as well as financially for institutions.^12^ There are several areas of intervention that potentially could have decreased the number of absences, despite the large increase in new COVID-19 patients. These include patient isolation and PPE strategies, patient and HCC testing protocols, institutional policies on HCC exposures, and travel restrictions.

Figure 2 depicts HCC absenteeism during March 2020 with a superimposed timeline of significant events during this month. Total HCCs absent are represented in gray. New patients diagnosed with COVID-19 in the ED are represented in black per the day in which the patient was tested in the ED, which was not always the day in which their test resulted. The blue scatterplot trend line represents the date that HCCs who were not wearing full PPE were exposed to patients with COVID-19. There was typically a discrepancy between date of exposure and date of absence given the delay in patient test results. The yellow scatterplot trend line represents new symptom development concerning for COVID-19 in HCCs. Some of those HCCs were initially asymptomatic after an exposure and then went on to develop symptoms. The green scatterplot trend line represents new HCC absences per day secondary to either not wearing full PPE with exposure to a patient with COVID-19 or symptom development concerning for COVID-19.

During the early phases of the pandemic, the presumption was that there was not substantial community transmission. It was believed that suspected patients could be screened and appropriately grouped based upon symptoms, travel, and exposure history. What resulted was a cohort-based PPE approach where HCCs used full PPE only when treating higher risk patients. As COVID-19 cases increased, it became difficult to adequately screen patients based on what was an evolving symptom profile alone, especially critically ill patients who were initially triaged to resuscitation rooms. Concurrently and as the increased disease prevalence became evident, a more targeted testing strategy (Figure S1) rapidly expanded to encompass a broader group of patients (Figure S2), which led to variability in testing. Patients who were not identified and tested during an initial HCC encounter, and thus were not initially evaluated in full PPE, were sometimes tested by a subsequent HCC and found to be positive.

Due to the initial cohort-based PPE strategy and the variability resulting from the evolving testing strategy, 80% of HCCs with exposure-related absences were exposed on March 13 and 14, two days following the broadening of testing criteria on March 12. After implementing a robust, triage testing protocol where full PPE was required for every patient encounter and testing was performed preceding patient rooming in the main ED, there were no more exposure-related HCC absences despite continued increases in COVID-19 diagnoses (Figure 2). The substantial initial number of HCC absences may have been prevented with broad and consistent use of full PPE during the early stages of the pandemic, with transition to a more targeted approach after the disease presentation and local community prevalence was better quantified.^13^–^19^

Many of the early absences described were in asymptomatic HCCs who had an exposure. Initially, our institutional policy dictated all exposures without full PPE required a 14-day home quarantine. This accounted for 39% of the total absences (Figure 1). The policy was revised on March 20 allowing for asymptomatic HCCs to return to work with a mask. This resulted in a brisk decrease in total HCC absenteeism (Figure 2). Anticipating and planning for extra HCC coverage at institutions that have implemented more conservative return-to-work policies may ensure adequate HCC staffing.

The institutional policy regarding testing HCCs was quite variable over time given testing shortages. Initially, only those HCCs who were symptomatic were tested, but the implementation of this policy remained somewhat variable. Most COVID-19 tests for HCCs took days to result. The protocol for HCC testing initially categorized tests for HCCs as outpatient tests, and often did not prioritize them over inpatient tests. Waiting for a negative test result caused delays in return to work for many HCCs. Implementing rapid testing that prioritizes HCCs may decrease days absent overall.^20^ Others have suggested implementing more frequent temperature checks for employees, as well as creating teams of clinicians who always work the same shift to minimize interactions between teams.^21^

Of those who required an absence, 39% had engaged in domestic or international travel, and all were symptomatic. Interestingly, 80% of the HCCs who tested positive for COVID-19 had a travel history (Figure 1). Early HCC travel restrictions could lessen the number of HCC absences due to travel-associated transmission during pandemics.

## LIMITATIONS

As a descriptive, retrospective study constructed from a database created for administrative purposes, the study is prone to selection bias and confounding variables. This limitation restricts the ability to determine causation. Additionally, the level of PPE compliance varied and, therefore, effectiveness cannot definitively be commented on. Community exposures were not tracked and may have contributed. Finally, the study was performed at a single center and may lack generalizability.

## CONCLUSION

This study describes a large number of healthcare clinician absences that occurred during a COVID-19 surge. It identifies several possible interventions that could help decrease the number and duration of these absences. Broad and consistent use of full PPE and appropriate return-to-work guidelines for asymptomatic, exposed HCCs may reduce virus exposures, new absences, and total days absent. Prioritizing HCC testing and implementing HCC travel restrictions should be further explored as possible associated factors. These techniques may be used by other frontline departments to anticipate and limit HCC absenteeism during this pandemic and in future similar events.

## Supplementary Information
