# Supplementary material for: Clinician Absences and Contributing Factors During a COVID-19 Surge: Potential Areas for Intervention and Planning
Source: West J Emerg Med. 2022 Feb 14;23(2):124–8. doi: 10.5811/westjem.2021.11.52715 (PMC8967457; doi:10.5811/westjem.2021.11.52715)
Supplement: Supplementary file 1 [file wjem-23-124-s001.docx]

**Figure S1.** Initial testing criteria published by our institution and used in the emergency department in accordance with national standards.

|  | Clinical Features | & | Epidemiologic Risk |
| --- | --- | --- | --- |
| 1 | Fever^1^ **or** signs/symptoms of lower respiratory illness (eg, cough or shortness of breath) | AND | Close contact^3^ with a laboratory-confirmed COVID-19 patient or sick person suspected of having COVID-19 |
| 2 | Fever^1^ **and** signs/symptoms of a lower respiratory illness (eg, cough or shortness of breath) requiring hospitalization | AND | A history of travel from affected geographic areas^5^ (China, Iran, Italy, Japan, South Korea) within 14 days of symptom onset |
| 3 | Fever^1^ with severe acute lower respiratory illness (eg, pneumonia, ARDS) requiring hospitalization^4^ and without alternative explanatory diagnosis (eg, influenza, RSV, and bacterial pneumonia)^6^ | AND | No source of exposure has been identified |

*ARDS,* acute respiratory distress syndrome; *RSV*, respiratory syncytial virus; *COVID-19,* coronavirus disease 2019.

**Figure S2.** Revised testing criteria published by our institution and implemented in the emergency department on March 12.

| Clinical Features | Risk Factors | SARS-CoV-2 Testing? |
| --- | --- | --- |
| Asymptomatic |  | No |
| Mild respiratory illness (fever, new cough, myalgias) not requiring hospitalization | - Age ≤65 years - No underlying medical conditions (see below), not pregnant or immunocompromised - Travel to affected areas^1^ within 14 days - Close contact with a confirmed COVID-19 case^2^ within 14 days | Testing is not recommended at this time. Mildly ill patients should be encouraged to stay home and practice social distancing while symptomatic |
| Mild respiratory illness (fever, new cough, myalgias) not requiring hospitalization | - Age ≥65 years - Pregnant - Chronic medical conditions (diabetes, heart disease, chronic lung disease, hypertension, cancer) - Immunocompromised (transplant, advanced HIV, or receiving immunosuppressive medications - Healthcare workers - Skilled nursing facility resident | Yes^3^ |
| Moderate respiratory illness (cough and shortness of breath) | - Age ≥65 years - Underlying medical conditions, immunocompromised, pregnant (see above) - Travel to affected areas^1^ or had close contact with a confirmed COVID-19 case^2^ within 14 days - Healthcare workers - Skilled nursing facility resident | Yes^3^ |
| Moderate-severe respiratory illness (eg, pneumonia, ARDS) requiring hospitalization | Known exposure or travel history not required | Yes^3^ |

*SARS-CoV-2,* severe acute respiratory syndrome coronavirus 2; *HIV*, human immunodeficiency virus; *ARDS*, acute respiratory distress syndrome; *COVID-19*, coronavirus disease 2019.
